# Efficient Water-Assisted Glass Cutting with 355 nm Picosecond Laser Pulses

**DOI:** 10.3390/mi13050785

**Published:** 2022-05-18

**Authors:** Edgaras Markauskas, Laimis Zubauskas, Bogdan Voisiat, Paulius Gečys

**Affiliations:** Center for Physical Sciences and Technology, Savanoriu Ave. 231, LT-02300 Vilnius, Lithuania; laimis.zubauskas@ftmc.lt (L.Z.); bogdan.voisiat@ftmc.lt (B.V.); paulius.gecys@ftmc.lt (P.G.)

**Keywords:** picosecond laser, glass cutting, characteristic strength, surface roughness, hatch

## Abstract

In this study, the cutting of borosilicate glass plates in ambient air and water with a 355 nm wavelength picosecond laser was carried out. Low (2.1–2.75 W) and high (15.5 W) average laser power cutting regimes were studied. Thorough attention was paid to the effect of the hatch distance on the cutting quality and characteristic strength of glass strips cut in both environments. At optimal cutting parameters, ablation efficiency and cutting rates were the highest but cut sidewalls were covered with periodically recurring ridges. Transition to smaller hatch values improved the cut sidewall quality by suppressing the ridge formation, but negatively affected the ablation efficiency and overall strength of glass strips. Glass strips cut in water in the low-laser-power regime had the highest characteristic strength of 117.6 and 107.3 MPa for the front and back sides, respectively. Cutting in a high-laser-power regime was only carried out in water. At 15.5 W, the ablation efficiency and effective cutting speed per incident laser power increased by 16% and 22%, respectively, compared with cutting in water in a low-laser-power regime.

## 1. Introduction

Glass is one of the most widely adopted engineering materials, preferred for its mechanical strength, chemical inertness, high thermal stability, and electrical resistance [1,2]. It is widely used in multiple areas, ranging from mass consumer electronics to specific applications, such as microfluidics, photonics, or microelectromechanical systems (MEMSs) [2,3,4].

Among different glass fabrication methods, glass cutting remains one of the most common and fundamental operations [5,6]. Mechanical glass cutting is typically performed by scoring and breaking, which produces large micro-cracks and splinters [5]. As a result, defects inflicted during cutting reduce the strength of the glass element [7], which ultimately could lead to glass shattering.

Nevertheless, the requirements for faster processing, higher precision, excellent surface finish, and tighter-than-ever tolerances are constantly increasing. For this reason, one of the most promising and versatile techniques for the drilling, milling, and cutting of fine features in the glass is direct laser ablation. Direct laser ablation enables the possibility of seamlessly switching between the machining of different types of features in glasses, unlike well-established mechanical methods. Furthermore, the laser beam can be sharply focused, allowing the production of micrometre-sized features with very high accuracy. Additionally, the laser process is flexible, highly repeatable, and easily automated [8,9]. The technology allows the cutting of features with a complex shape, consisting of inner and outer contours [10]. Opaque and highly absorptive glasses can be processed, unlike other common laser-based techniques, such as rear side ablation [11] and the internal scribing approach [9,12].

The transition from continuous-wave and short (nanosecond) pulses to ultrashort lasers reduces thermal damage in glasses [13]. Furthermore, the dominant mechanism causing ablation shifts from avalanche ionisation to multiphoton ionisation for pulses shorter than 50 ps [14]. This improves the localisation of absorbed laser energy and reduces thermal damage. The avoidance of such defects is crucial for cutting and scribing applications because they determine the strength of the machined element [7,15]. Therefore, laser ablation with ultrashort pulses has become an attractive method for the precise machining of thin glasses (<1 mm) [15].

The average laser power of modern ultrashort lasers is constantly growing, allowing one to achieve higher-than-ever processing speeds. Thus, with decent (industrially accessible) laser focusing, such systems can easily surpass optimal laser fluence levels for most engineering materials. In order to utilise full laser potential at optimal material processing conditions, one must either increase the laser beam spot size at the expense of machining accuracy or increase the laser pulse repetition rate to maintain optimal fluence levels. Unfortunately, excessive heat would accumulate in either case and ultimately lead to fractures and destruction of the brittle material even for ultrashort pulses [16,17].

Fortunately, excessive heat which is generated during the laser processing can effectively be taken away by introducing liquid into the laser ablation zone [16,18,19,20,21]. As a result, brittle material can withstand higher thermal loads, and higher laser power can be irradiated into the workpiece, increasing the process throughput. Additionally, multiple studies have reported the improved extraction of ablation products, reduced plasma shielding, and the generation of high-pressure mechanical shockwaves, which further contributed to material removing rates [22,23]. Additionally, improved machining quality was also observed: reduced heat-affected zones (HAZs), micro-cracks, and the re-deposition and recast of ablative debris [21,24,25,26,27].

Water is most commonly used as a laser ablation assisting liquid since it is cheap, harmless, and recyclable [20,28]. Water can be introduced into the ablation zone in multiple ways: by submerging the workpiece into standing [22,26,29,30] or flowing water [20,21,31,32]; by guiding a laser beam in the water jet [33,34]; or by spraying water mist [18] or a water jet [19,28] next to the laser beam. Most studies have investigated laser ablation with workpieces submerged into a standing or low-velocity liquid flow with a liquid layer thickness of a few to tens of millimetres. The key limitations of such a design were noted in [20,28,35]: laser heating formed bubbles around the ablation zone, generating waves at the surface of the liquid. Consequently, waves caused instability in laser processing conditions—variations in laser focus position, beam diffraction, and refraction angles; as a result, hampering the continuity and uniformity of ablated grooves [35]. Furthermore, Tangwarodomnukun et al. [35] reported a substantial 6.5% loss of laser power in a 2 mm thick water layer for 1080 nm wavelength radiation, whereas Kruusing et al. [36] observed the laser (heating) power loss due to the water cooling.

Fortunately, air–water interface instability and laser absorption in the water layer can be addressed by employing a thin and flowing water film [16,18]. This can be achieved by spraying a water mist into the ablation zone instead of submerging the workpiece into the liquid. Transitioning to shorter laser wavelengths can further reduce laser radiation absorption in the water layer. For example, absorption of the 355 nm wavelength radiation is about 300 times lower than the absorption of the widely used 1064 nm wavelength [36]. The highest absorption length in water is reportedly in the range of 400–600 nm, with the peak value at 500 nm [36]. However, transitioning to UV wavelengths can improve laser energy coupling in glasses [14] and localise laser-induced damage into a smaller area [13]. For 355 nm, higher ablation efficiency and lower ablation thresholds compared with 532 and 1064 nm wavelengths were reported in [14]. As a result, glass chipping and microcrack formation can be further reduced, improving cut edge strength and making glass more resistant to tensile stresses.

In our previous study, we demonstrated the efficient milling and cutting of borosilicate glasses using a picosecond laser working at a 1064 nm wavelength in a water-assisted environment [16]. Water introduction into the ablation zone significantly improved effective glass cutting speeds and the morphology of the cut wall compared with glass ablation in ambient air.

As stated above, the transition to a shorter wavelength could further improve the cut quality due to better laser energy coupling, improving the strength of the laser-cut glass elements. To the best of our knowledge, no research involving efficient borosilicate glass cutting with UV picosecond pulses in a water-assisted environment has been conducted thus far. Additionally, we investigated the impact of the hatch distance on the cut wall quality and the characteristic strength of laser-cut glasses when the cut line consisted of multiple lines scanned in parallel.

Here, we experimentally studied the cutting of 0.4 mm thick borosilicate glass plates with a picosecond laser working at 355 nm radiation wavelength. Glass ablation was carried out in ambient air and water using optimised laser processing parameters for efficient glass ablation. The glass shattered at elevated laser powers (above 4 W) in ambient air; therefore, low (2.1–2.75 W) and high (15.5 W) average laser power cutting regimes were investigated separately. The cutting quality was evaluated using an optical microscope and a profiler. Four-point bending tests were conducted to determine the bending strength of laser-cut glass strips.

## 2. Materials and Methods

Glass cutting experiments were performed using a picosecond laser: Atlantic from Ekspla. The emission wavelength was 355 nm, the pulse width was 10 ± 3 ps, and the maximum average laser power at the sample’s surface was 15.5 W. The laser pulse repetition rate was adjustable between 0.4 and 1 MHz. The emitted light intensity profile was similar to Gaussian and had linear polarisation. Cutting experiments were carried out using P polarisation only (the polarisation vector was perpendicular to the laser beam scanning direction).

The optical setup consisted of a laser, a beam expander, mirrors to direct the laser beam to the scanning system, and a focusing lens. The laser beam movement was controlled with a galvanometer scanner: IntelliSCAN_de_14 from ScanLab. For focusing, we used an f-theta lens with a focal distance of 100 mm. Laser beam spot sizes were measured using Liu’s method [37]. At the focal position, the minimal diffraction-limited spot size (diameter) was 15 µm with a beam expander installed in the laser beam path and 30 µm without the expander. The laser fluence *F* (values reported further in the text) was evaluated using the expression $F=2E/\left(\pi r^{2}\right)$, where *E* is the laser pulse energy and *r* is the radius of the focused laser beam. The focal plane was set on top of the glass sample for the laser fluence and spot size measurements. For glass cutting experiments, the laser beam focal point was shifted 200 µm below the surface of the glass sample.

In this study, we used borosilicate glass plates (D263m) with a thickness of 400 µm. The length and the width of the glass plates were 26 mm and 20 mm, respectively. Glass plates were cleaned before the laser cutting with high-purity acetone. Cutting quality was analysed with an optical microscope: Eclipse LV100NDA from Nikon. The cut sidewall topographies were recorded with the S neox optical profiler from Sensofar.

Morphology and topography analyses were followed by glass cleaving experiments to determine the characteristic strength of laser-cut samples. For this, glass plates were cut into rectangular 26 × 6 mm^2^ glass strips. The bending force was measured with an Alluris FMI-S30A5 dynamometer.

**Figure 1:**
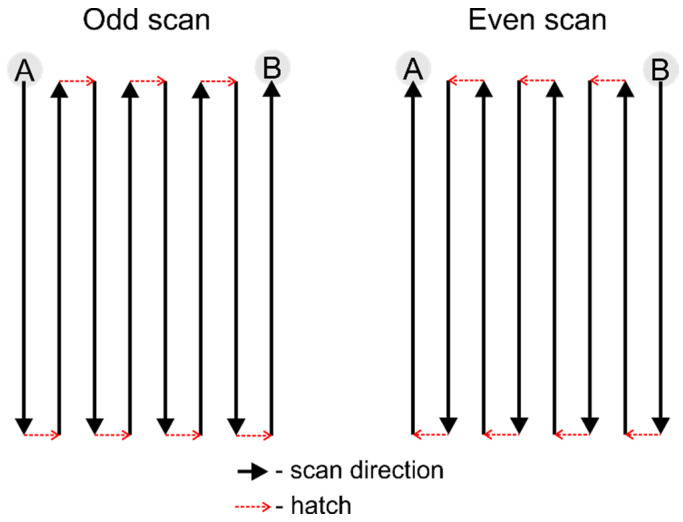
Graphical representation of the scan geometry used for cutting glass.

The laser scanning geometry used for cutting the borosilicate glass is presented in Figure 1. Multiple lines were scanned in parallel, separated by the hatch (the distance between two scanned lines). The number of lines in a single scan defined the cut’s width. After each scan, the hatch direction was changed to the opposite. In the case of an odd scan, the scanning started at position A and ended at position B. An even scan started where the odd scan had ended (position B) and returned to position A by following the same scan path backwards.

**Figure 2:**
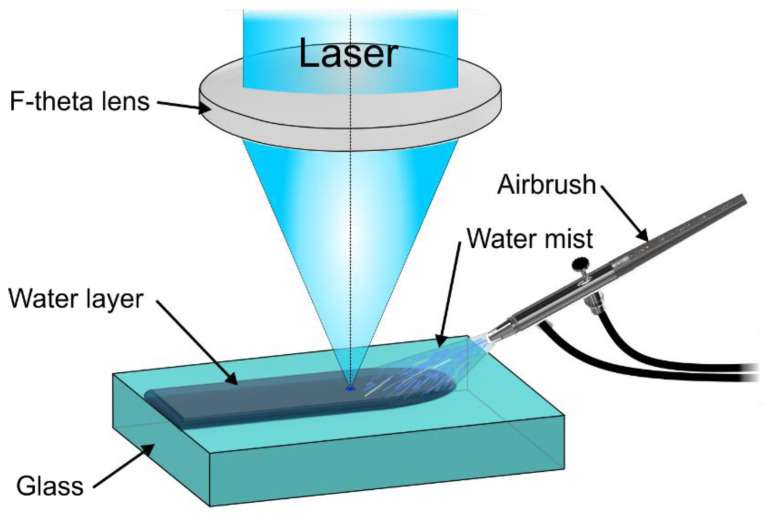
Experimental setup. Airbrush sprayed water mist onto the glass surface, forming thin and flowing water film.

A thin flowing water film was formed at the surface of the glass workpiece by spraying water mist (see Figure 2). The mist was formed with a commercially available airbrush connected to a pressurised air (3 bar). Here, high-velocity air atomised water into droplets which then were sprayed onto the glass surface and formed a liquid film flowing through the laser ablation zone. The liquid layer was the thickest at the beginning and gradually thinned out as the distance from the nozzle tip increased. In this study, the liquid film flow direction was parallel to the laser scanning direction. At the start of the cut line, the thickness of the liquid layer was 750 µm, whereas at the end of the cut line (26 mm away), the thickness decreased to 450 µm. Variation in layer thickness had no significant effect on ablation efficiency over the length of the cut.

**Figure 3:**
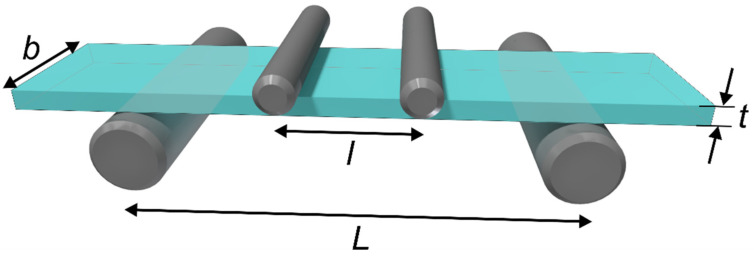
The four-point bending test setup.

A four-point bending test was performed to assess the characteristic strength of glass strips cut with a laser. The bending test setup is presented in Figure 3. The testing setup consisted of two inner loading rollers with an inner span of 6 mm and two support rollers with a span of 16 mm. The diameter of loading and support rollers was 2 and 6 mm, respectively. During the load, the side of the glass sample in contact with support rollers was under the tensile load, whereas the opposite side was under the compressive load. Both sides underwent a different type of load during a test; therefore, bending tests were conducted on both sides of glass samples. The loading rate was 1.7 MPa/s.

The maximum bending strength σ is given by the following formula [38]:

(1) $$\sigma =\frac{3F\left(L-l\right)}{2bt^{2}},$$

where *F* is the load at glass sample failure, *b* is the width, *t* is the thickness of the sample, and *l*, *L* are the distance between the loading and supporting rollers, respectively.

Glass is a brittle material that usually presents a widely scattered strength. Some samples cut under the same processing parameters may break under light load, whereas others will withstand significant bending forces [39]. For this reason, Weibull cumulative distribution is often employed to describe the distribution of the characteristic strength of such materials [40]:

(2) $$P_{f}\left(\sigma \right)=1-exp{\left(-\frac{\sigma }{\sigma_{0}}\right)}^{m}$$

Here, *σ*_0_ and *m* are the characteristic strength and shape parameters of the Weibull modulus. Characteristic strength defines the bending strength at which 63.2% of all samples fail, whereas the shape parameter indicates the dispersion [39], and $P_{f}$ is the fracture probability.

During tests, a force was applied on laser-cut strips. The maximum bending strength of each strip was calculated based on the measured *F* at which glass failure occurred. Obtained σ values were ranked in ascending order, *i* = 1,2,…,*n*. Probability of fracture should be assigned to every calculated $\sigma_{n}$, where $0<P_{f}<1$, but because the exact probability values for each $\sigma_{n}$ is not known, an estimator was used to find these values [41]:

(3) $$P_{f,\;i}\left(\sigma \right)=\frac{\left(i-0.5\right)}{n}.$$

This is one of the most commonly used estimators and preferred one for smaller sample sizes of fewer than 50 measurements [42]. Finally, Equation (3) was fitted with Weibull cumulative distribution (Equation (2)) to extract σ_0_ and *m* parameters.

## 3. Results and Discussion

### 3.1. Low-Laser-Power Cutting Regime

#### 3.1.1. Cutting Process Optimisation

Thin glass plates are sensitive to thermal stresses caused by laser ablation and can easily shatter when higher laser power is irradiated into the material [16]. Glass plates used in this research fractured in ambient air if incident laser power exceeded 4 W. For this reason, glass cutting experiments were split into two parts: low and high (average) laser power regimes. This section presents laser parameter optimization for efficient borosilicate glass cutting at low laser power (<4 W). In Section 3.2, results on high-laser-power cutting are presented.

Firstly, laser ablation parameters for cutting borosilicate glass in ambient air and water were optimised. Initial (not optimised) ablation parameters were the same for both cutting environments: *ν* = 400 kHz (laser pulse repetition rate), *V* = 200 mm/s (laser beam scanning speed), *h* = 4 µm (hatch), and *P*_avg_ = 4 W (average laser power). The length and width of the optimisation cuts were 5 and 0.3 mm, respectively. During the optimisation step, a number of parameters were optimised: laser fluence, laser beam scanning speed, pulse repetition rate, and the hatch. For every laser parameter set, a minimum number of laser scans was found for a complete and consistent cut-through.

**Figure 4:**
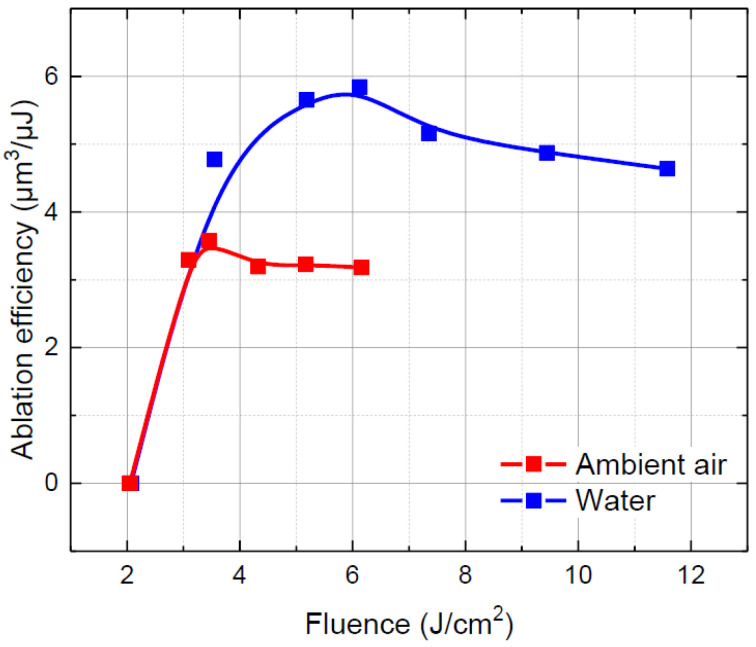
Ablation efficiency versus laser fluence in ambient air and water. Dots are connected to guide the eye.

First, the laser fluence was optimised in both environments. The laser fluence was varied from 2 to 12 J/cm^2^ by changing the laser pulse energy. The results are presented in Figure 4. The shapes of both ablation efficiency curves were similar, despite different ablation environments. However, the peak of the ablation efficiency curve in water was higher by 63.6% (3.57 versus 5.84 µm^3^/µJ) and shifted towards higher fluencies (from 3.5 J/cm^2^ to 6.1 J/cm^2^).

After the optimal laser fluence was found, the laser pulse repetition rate was re-adjusted, keeping in mind that the average laser power should be maintained below 4 W to avoid glass fracture in ambient air. Laser wavelength conversion efficiency from 1064 nm (fundamental wavelength) to 355 nm (third harmonic of the fundamental wavelength) depends on the pulse intensity. Thus, increasing the pulse repetition rate above 400 kHz and keeping the pump level fixed resulted in a slight decrease in laser output power.

With that being said, further optimisation in water was carried out at 529 kHz and the average laser power of 2.75 W, whereas optimisation in ambient air was conducted at *ν* = 653 kHz and *P*_avg_ = 2.1 W. Laser fluence was maintained optimal in both environments, despite slightly different average laser power levels.

**Figure 5:**
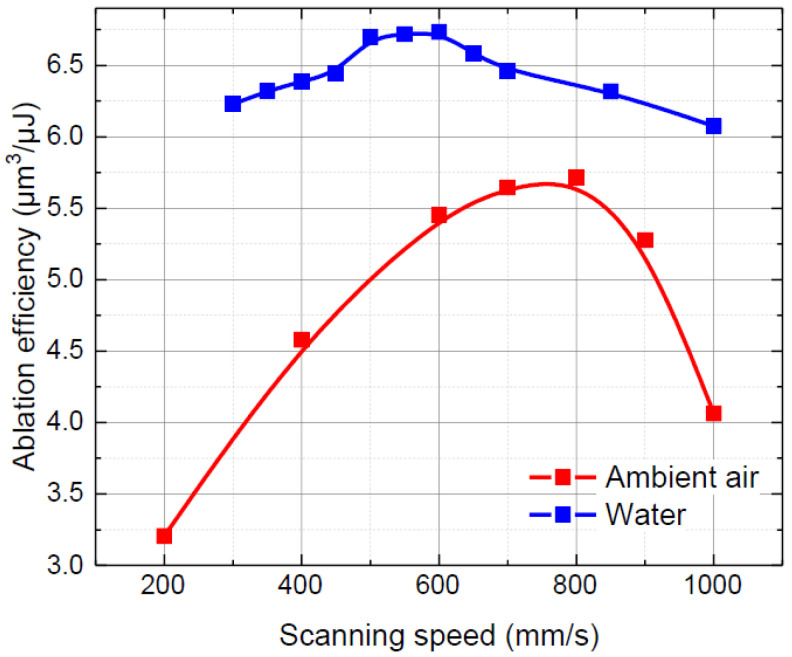
Ablation efficiency versus the laser beam scanning speed in ambient air and water. Dots are connected to guide the eye.

Then, the laser beam scanning speed was varied from 200 to 1000 mm/s (see Figure 5). Glass ablation in water remained more efficient in the investigated scanning speed range. Additionally, varying the scanning speed outside the optimal values in water was not as detrimental as in ambient air. The optimal laser beam scanning speed for glass ablation in water was lower (600 mm/s) compared with ablation in ambient air (800 mm/s). Here, the difference in the optimal scanning speed was determined by the different pulse repetition rates in both environments.

A decrease in ablation efficiency at scanning speeds below optimal in ambient air was associated with increasing laser beam shielding with plasma and debris due to heat accumulation, whereas ablation efficiency loss at scanning speeds above optimal was associated with decreasing temperature in the ablation zone from the optimal due to the increasing distance between laser pulses [43,44].

**Figure 6:**
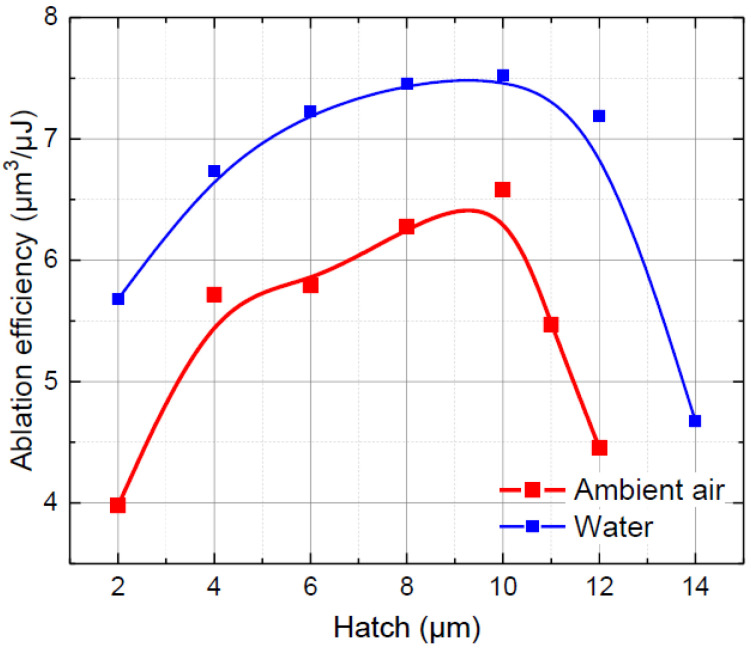
Ablation efficiency versus hatch distance in ambient air and water. Dots are connected to guide the eye.

Finally, a hatch distance was optimised. The distance between the scan lines was varied between 2 and 14 µm to find an optimal hatch value. Additionally, the number of parallel cut lines was adjusted to maintain a constant cut width. Ablation efficiency dependence on the hatch distance is presented in Figure 6. Here, the peak efficiency was obtained at the same hatch distance (10 µm) in both environments, which indicated that the hatch distance mostly depended on the laser beam spot size, but not on the processing environment. At a 10 µm hatch distance, the laser ablation efficiency in ambient air was 6.6 µm^3^/µJ. The ablation efficiency in water was 14% higher, reaching 7.5 µm^3^/µJ.

After the optimised laser parameters were determined, we varied the laser fluence, pulse repetition rate, laser beam scanning speed, and hatch again (only in a narrower parameter range). Despite this, the parameter values yielding the highest ablation efficiency remained unchanged, indicating the optimal glass cutting parameter set.

Therefore, the final (optimised) processing parameters for cutting 0.3 mm wide cuts in a low-laser-power regime are presented in Table 1. Under optimised glass ablation parameters, the effective cutting speed of 0.4 mm thick glass plates in water was 0.26 mm/s. Cutting in ambient air was slower—0.19 mm/s. The difference in effective cutting speed mostly resulted from the difference in incident laser power between two environments (2.75 W in water and 2.1 W in ambient air). Considering this, the effective cutting speed per incident laser power (W) was 0.095 mm/s/W in water and 0.09 mm/s/W in ambient air.

Despite the 14% higher ablation efficiency in water-assisted conditions, the improvement in effective cutting speed per Watt was modest (5.5%). The reason for the discrepancy between the ablation efficiency and effective cutting speed was caused by the steeper cut walls produced in water [16]. Strong shockwaves are created during laser ablation in a liquid environment that impinge on the cut sidewalls, producing shallower taper angles [16]. As a result, a larger volume of material was removed, yielding a higher ablation efficiency, despite removing the same material layer thickness per laser scan.

#### 3.1.2. Cut Sidewall Quality

The hatch (*h*) is the distance between two scanned lines that defines the degree of laser beam overlap between two neighbouring cut lines. The effect of hatch distance on the bottom of milled cavities has been investigated in multiple papers [44,45,46,47]. However, we could not find extensive research on the influence of the hatch on the quality of laser cutting.

**Figure 7:**
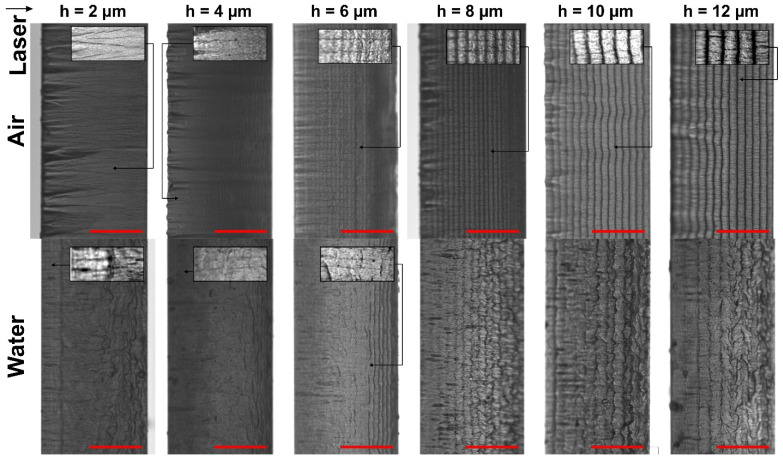
Optical micrographs of cut sidewalls. Cases for cutting in ambient air and water at different hatch values are presented. Scale bars represent 0.2 mm and apply to all panels in this figure. The laser beam entry side is indicated with an arrow.

For this reason, rectangular glass strips (26 × 6 mm^2^) were cut out of the larger glass plates using the optimised laser parameter sets presented in Table 1 (2.1 W in ambient air and 2.75 W in water). The optimal hatch in both environments was 10 µm. Nevertheless, we produced additional cuts with the following hatch values: 2, 4, 6, 8, and 12 µm. Here, only the hatch value was varied, whereas other parameters remained unchanged. Micrographs of laser-cut walls are depicted in Figure 7.

Cut sidewalls were covered with periodically recurring ridges. These formations were parallel to the laser beam scanning direction and spanned uninterrupted throughout the length of the cut. Ridges are visible as the dark lines in Figure 7 separated by lighter areas—the concavities.

**Figure 8:**
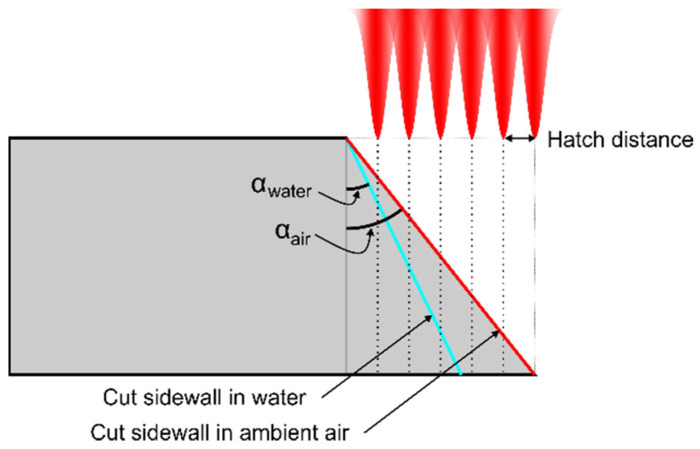
Schematic of the side view of the glass plate. Above the glass plate, a cumulative laser intensity profile is shown. Dashed lines (normal to the glass surface) extrapolated from the cumulative laser intensity peaks indicate positions where concavities were formed on the inclined cut wall. Ridges were formed between the concavities.

In the experiments, we used a Gaussian laser beam intensity profile. Thus, these formations resulted from the cumulative beam intensity distribution projected onto the glass plate after multiple laser scans (Figure 8). The accumulated laser intensity distribution (the distance between intensity minimums and maximums) depended on the hatch distance between individual cut lines. Concavities represent the laser beam intensity peaks, where more material was removed. In Figure 8, dashed lines (normal to the glass plate surface) were extrapolated from the laser intensity peaks to the inclined cut sidewall, indicating positions where concavities would be formed. Ridges were formed in intensity minimums between the cut lines, thus removing less material. However, as the hatch distance decreased to 2 µm in ambient air and 4 µm in water, the ridges became indistinguishable to the eye. Hatch became comparable to the pitch, yielding a relatively uniform overlap in both directions.

**Figure 9:**
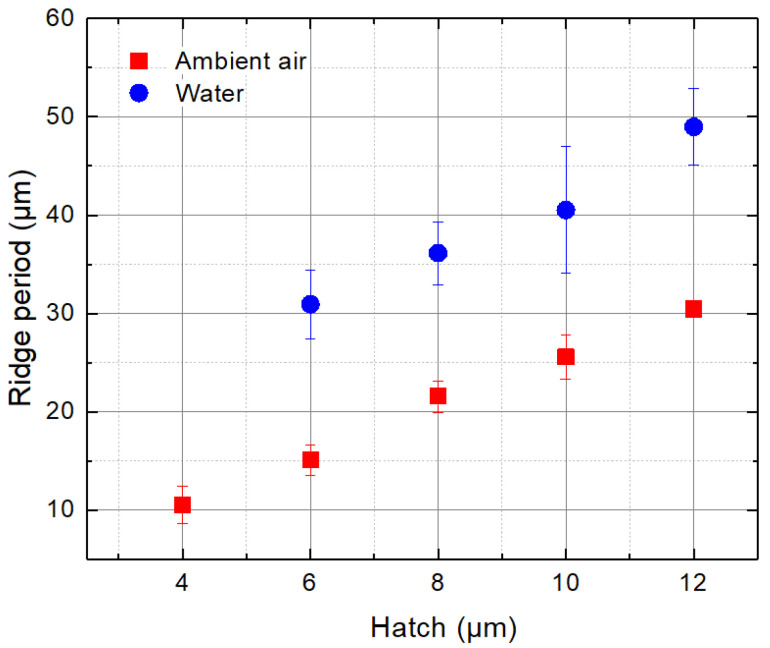
The period of ridges versus the hatch distance.

The period of ridges *H* depended on the hatch distance and the cut wall taper angle: sin(α)=h/H (see Figure 8), where *α* is the taper angle obtained in water (*α*_water_) or in ambient air (*α*_air_). According to the measurements, the average taper angle in water was 16°, whereas the angle in ambient air was larger—23.4°. As a result, fewer ridges were projected on glass strips that were cut in water, resulting in larger ridge periods. The period of ridges versus the hatch distance is presented in Figure 9. At *h* = 6 µm, the ridge period in ambient air was 15.1 µm, whereas in water, it was almost two times larger (30.9 µm). The ridge period increased more quickly in water with the hatch. As a result, the difference in periods gradually increased to almost 20 µm at *h* = 12 µm.

In both cutting environments, cut wall steepness decreased slightly near the bottom of the cut. This resulted in a denser ridge formation in that area. Nevertheless, the relationship between the ridge period, taper angle, and hatch distance remained valid.

The waviness of ridges seen in Figure 7 in ambient air was caused by glass chipping at the front surface of the glass plate. Chips originated at the front glass surface and propagated more deeply into the glass plate during the cutting, forming oblong concavities perpendicular to the laser beam scanning direction. The dense chipping at the cut edge is a characteristic of glass ablation in ambient air due to heat accumulation [16]. Vertical oblong concavities tended to grow with the decreasing hatch distance. This indicated increasing heat accumulation [48].

In the case of water-assisted cutting, chipping at the cut edge and the consequent formation of oblong vertical concavities was suppressed by efficient cooling. However, ridges were wavy near the bottom surface of the cut sidewall due to laser beam distortion by the water layer. This effect was attributed to the laser beam disturbance in a liquid–vapour layer, which increased with the depth of the cut (water flow instability, formation and collapse of bubbles, liquid vaporisation [49,50,51]). According to the micrographs, glass melting was pronounced in distorted areas.

Additionally, we observed vertical cracks formed at the cut sidewall normal to the glass surface (a network of cracks spanned between the front and the back surfaces, as shown in the insets in Figure 7). Cracks were more visible on cut sidewalls produced in ambient air due to a much smoother cut wall surface (see insets at *h* = 2, 6, 8, 10, and 12 µm). In the case of cutting in water, a network of vertical cracks was visible in smoother areas of the sidewall where surface distortion was minimal. Here, cracks were clearly visible at *h* = 2, 4, and 6 µm. The melting was more pronounced in distorted areas of the cut sidewall. Thus, the network of cracks could have been covered with re-solidified melt, hindering their detectability [52]. However, we could not identify cracks in distorted areas in the micrographs presented in Figure 7.

At *h* between 6 and 8 µm, the direction of the cracks was strictly vertical in ambient air, propagating from the front surface towards the bottom of the glass strip. However, as the *h* decreased and ridges could not be distinguished any more (*h* = 2), the direction of propagating cracks became not as strict, allowing individual cracks to deviate up to an angle of 14° from the normal to the glass surface. In some cases, cracks intersected each other. Furthermore, the density of the crack network was highest at *h* = 2 µm, indicating the highest thermal damage. At the largest investigated hatches (10 and 12 µm), cracks became discontinuous and short and were prone to abruptly change direction between ridges.

#### 3.1.3. Cut Edge Quality 

A high edge quality is critical for the mechanical robustness of glass parts. Minimising glass chipping and microcrack formation at the cut edge reduces the loss of strength of machined glass elements [7]. Furthermore, such defects tend to grow under the tensile strength and, over time, could cause the fracture of glass elements [53].

We assessed the front and back surface edge quality in more detail for the following hatch distances:

Optimal hatch yielding the highest ablation efficiency (10 µm in ambient air and water);

Ridge-free hatch (2 µm in ambient air and 4 µm in water);

An intermediate hatch between the optimal and ridge-free hatch values (6 µm in ambient air and 7 µm in water).

Multiple studies have shown that band-like damage can occur at the back surface of transparent media during laser scribing and cutting [53,54,55,56], potentially hindering the mechanical robustness of brittle glass elements [55]. This damage is mainly associated with laser beam refraction and reflection from the ablated crater/channel walls and can be avoided, or at least minimised, by properly selecting the laser beam polarisation direction. In this study, we did not observe any formation of band-like damage next to a laser cut at P polarisation. For this reason, experiments were conducted using P polarisation only, and no other polarisation states were investigated.

A total of 48 strips were cut for every preselected hatch (24 in ambient air and 24 in water). Each strip was cut with two laser cuts along the longer edge of the glass plate. This way, four cut edges were inspected per single glass strip—two edges at the front and two at the back surface of each strip.

**Figure 10:**
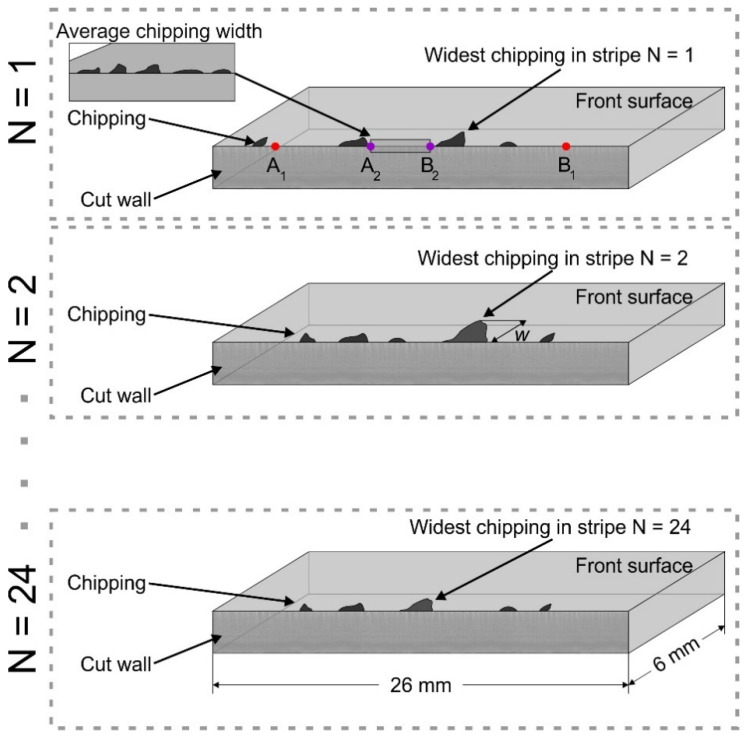
Schematics for evaluating the mean maximum and the average chipping widths at the cut edge. The mean maximum chipping width was evaluated over a distance of 10 mm (between points A_1_ and B_1_). An average chipping width was evaluated over a distance of 1 mm (between points A_2_ and B_2_).

We evaluated the cut edge quality of glass by assessing the mean maximum and the average chipping widths on both sides of laser-cut glass strips (see Figure 10). The mean maximum chipping width is defined as the width of the single widest chip per cut edge averaged over all strips cut under the same laser parameter set. The average chipping width (*w*) is an average cut edge deviation from the cut line due to glass chipping measured normal to the glass surface, as shown in Figure 10. The cut edge quality at the front and back surfaces was assessed separately. The mean maximum chipping and the average chipping widths were evaluated in a central part of the cut edge over distances of 10 and 1 mm, respectively.

**Figure 11:**
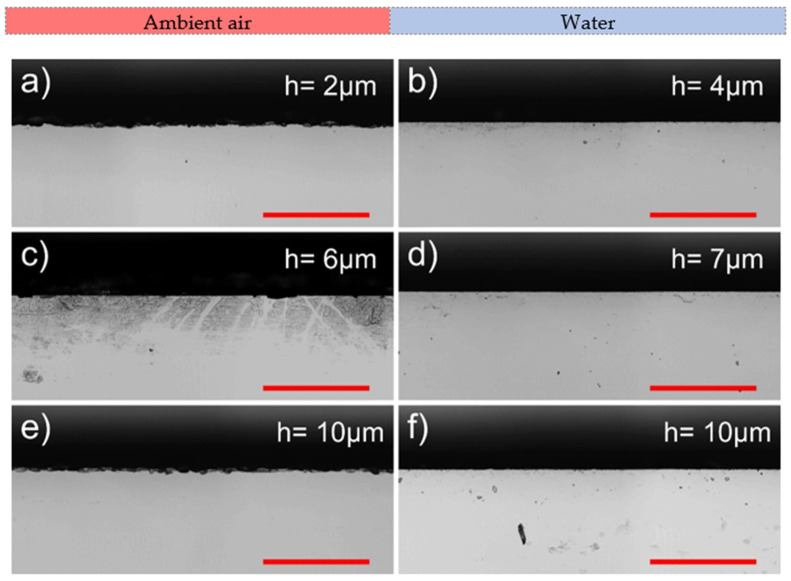
Optical micrographs showing cut edges at the front surface produced in ambient air (to the left) and water (to the right). Rows represent different hatch distances: ridge-free (**a**,**b**), intermediate (**c**,**d**), and optimal (**e**,**f**). The scale bars represent 200 μm and apply to all panels in the figure.

The most noticeable difference in cut edge quality between the two environments was observed at the front surface of the laser-cut glass strips (see Figure 11). Cutting in water was superior to cutting in ambient air in terms of cut edge quality. The average chipping width in water was 7.2 ± 1.2 times smaller than in air (see Table 2). Furthermore, variation in hatch distance in the water had an insignificant effect on the average chipping width, which was distributed between 0.75 and 0.85 µm. Here, the smallest value of 0.75 ± 0.35 µm was observed at the optimal hatch distance (*h* = 10 µm).

Cutting in ambient air produced rougher cut edges. The smallest value of 4.3 ± 1.8 μm was measured at the optimal hatch. Transition to smaller hatch values increased the average chipping width by up to 49% to 6.2–6.4 µm, depending on the hatch distance.

**Figure 12:**
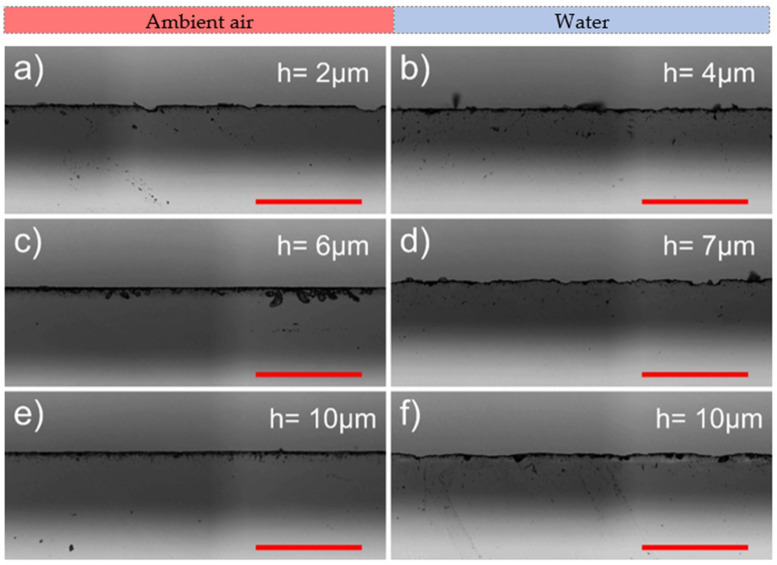
Optical micrographs showing cut edges at the back surface produced in ambient air (to the left) and water (to the right). Rows represent different pitch distances: ridge-free (**a**,**b**), intermediate (**c**,**d**), and optimal (**e**,**f**). The scale bars represent 200 μm and apply to all panels in the figure.

However, contrasting results were observed at the back surface (see Figure 12). Here, cutting in ambient air outperformed cutting in water by producing smoother cut edges. Furthermore, cut edges at the back surface were smoothest at the smallest hatch distance. In ambient air at *h* = 2 µm, the average chipping width was only 3.9 ± 2.9 µm. In water, at *h* = 4 µm, the average chipping width was 13% higher than in air (4.4 ± 2.2 µm). Unexpectedly, the highest average chipping width was observed at the optimal hatch distance (*h* = 10 µm) in both environments: in ambient air, the value was 6.7 ± 3.3 µm, whereas in water, it was even larger—6.9 ± 4.3 µm.

**Figure 13:**
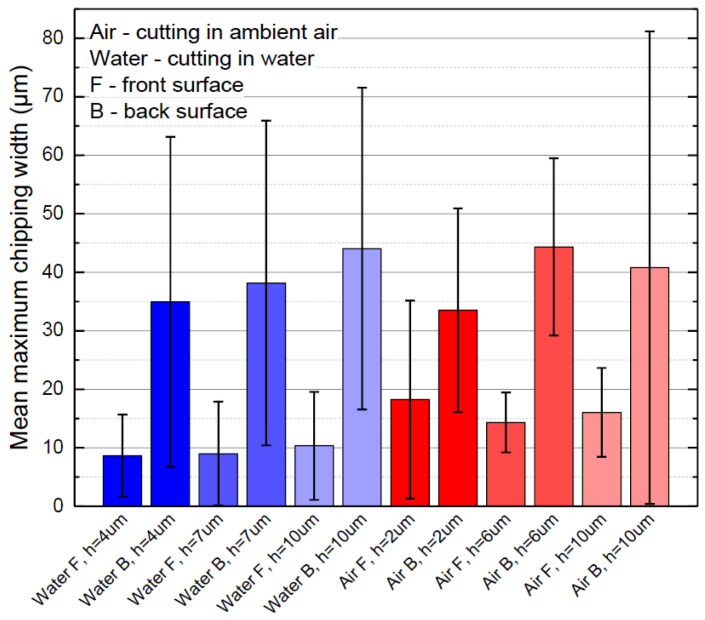
Mean maximum chipping width at the cut edge.

Next, we evaluated the mean maximum chipping widths at the back and the front surfaces. The formation of wide chippings that were significantly wider than the average chipping width was scarce. However, this could not be avoided entirely, even when cutting under optimal laser processing parameters. The mean maximum chipping width at the back surface was distributed between 34 and 44 µm in both cutting environments, with the smallest widths at *h* = 2–4 µm (see Figure 13). At the front surface, maximum chipping was smaller in water by 43% (9.3 ± 0.9 µm in water and 16.2 ± 2 µm in ambient air). Furthermore, error bars in Figure 13 reveal that the mean maximum chipping width was more consistent at the front surface than at the back.

#### 3.1.4. Cut Wall Roughness

**Figure 14:**
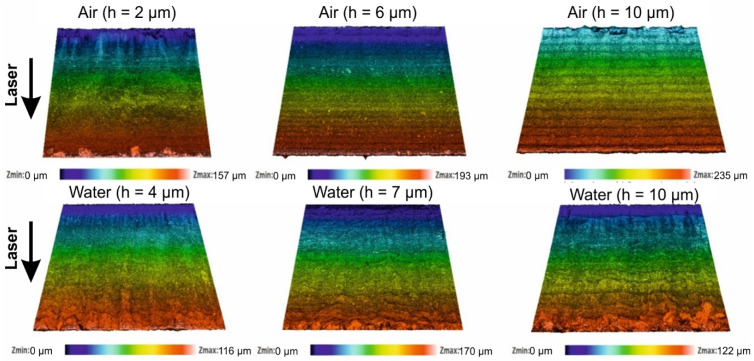
Topographies of cut sidewalls produced in ambient air and in water. Cases for three different hatch distances are presented (optimal, intermediate and ridge-free). Laser beam entry side is indicated with an arrow and applies to all panels in the figure.

A rougher sidewall could indicate the presence of larger defects which negatively affect the strength of glass strips [17]. For this reason, we studied the roughness of laser-cut sidewalls produced in ambient air and water at preselected hatch values (optimal, ridge-free and intermediate). Recorded 3D topologies at different *h* values are depicted in Figure 14.

**Figure 15:**
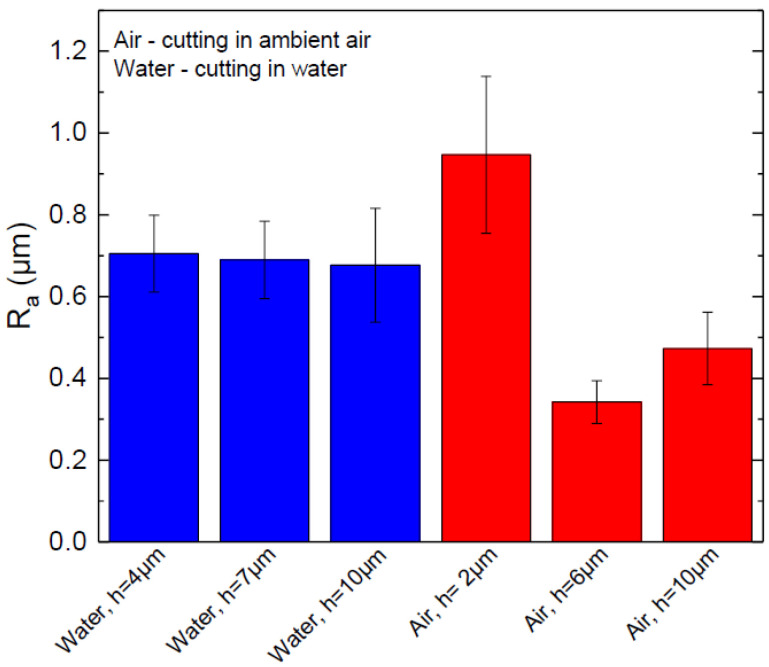
Cut wall roughness (*R_a_*) versus hatch in ambient air and water. Error bars indicate standard deviation.

The surface roughness (*R_a_*) was measured along the laser beam scanning direction 200 µm below the glass surface. Cutting in water produced a consistent cut sidewall surface roughness of *R_a_* = 0.69 ± 0.1 µm in the investigated *h* range (see Figure 15).

On the other hand, cut wall roughness varied greatly in ambient air. At the intermediate and optimal hatch values, cut sidewalls were smoother than in water. The overall smoothest sidewall of 0.34 µm was measured at *h* = 6 µm in ambient air. However, the maximum roughness of 0.95 ± 0.2 µm was also measured in the air at *h* = 2 µm. An abrupt increase in roughness at *h* = 2 µm was associated with the formation of vertical oblong concavities.

Improved glass cooling in water avoided cut sidewall quality degradation related to heat accumulation, showing a consistent surface roughness irrespective of the hatch value. The roughness was higher than in ambient air due to increased mechanical forces acting on the glass wall during the laser ablation (collapse of cavitation bubbles, confined plasma generated shockwaves), which led to a more mechanical glass erosion and porous-looking cut wall surface [16,17,57].

#### 3.1.5. Flexural Strength

**Figure 16:**
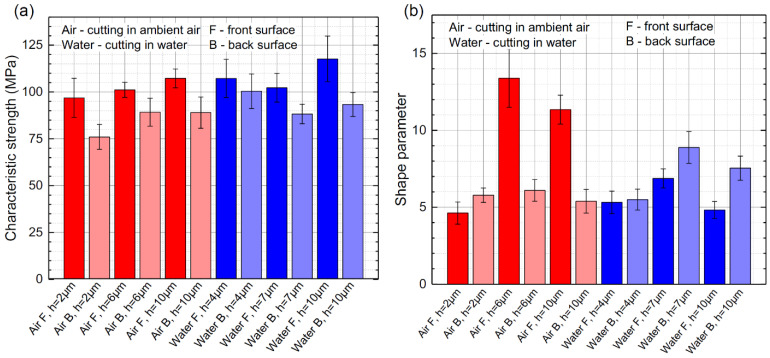
Characteristic strength (**a**) and shape parameter (**b**) when loading force is applied from the front and the back glass strip sides. Cases for cutting in ambient air and water are presented.

Laser-cut glass strips were broken using the four-point bending setup shown in Figure 3. The failure occurred at the tensioned side of the glass strip, facing support rollers. For this reason, we conducted bending tests on both sides of glass strips. We broke 12 samples per side for every preselected hatch value in both cutting environments. Extracted characteristic strength (the bending strength at which 63.2% of all samples fail) and shape parameters are presented in Figure 16. Glass strips were cut using average laser powers of 2.1 and 2.75 W in ambient air and water, respectively.

Glass strips cut in the water had higher strength than those cut in ambient air. The highest front side strength of 117.6 ± 12 MPa was recorded at the optimal hatch (*h* = 10 µm), whereas the highest back side strength was recorded at the ridge-free hatch value of *h* = 4 µm (101 MPa). Nevertheless, the strength at the back side at the optimal *h* value was only lower by 7 MPa compared with the maximum strength recorded at the back side.

For the front side bending in ambient air, characteristic strength increased with the hatch. The smallest characteristic strength of 96.8 ± 10 MPa was obtained at *h* = 2 µm, and reached the highest value of 107.3 ± 5 MPa at *h* = 10 µm. The lowest strength at the back side was also measured at the smallest hatch value of 2 µm (76 ± 7 MPa), but the strength increased to 89 ± 8 MPa at *h* = 6–10 µm.

Overall, the strength at the front side (surface) was higher than at the back, between 7% and 27%, depending on the *h* value and the cutting environment.

As noted in Section 2, the shape parameter is dimensionless and indicates the dispersion of the characteristic strength. Therefore, measurements with a higher *m* parameter are less scattered, giving more predictable and consistent results. For front side bending, the highest shape parameters were extracted in ambient air at intermediate and optimal hatch values. Although the characteristic strength was smaller in ambient air, the strength was more consistent at these hatch values. The highest *m* parameter for back side bending was obtained in glass strips cut in water at intermediate and optimal hatch distances (8.9 ± 1 and 7.5 ± 0.8). In other cases, the *m* parameter was relatively consistent, with an average value of 5.6 ± 0.7.

Characteristic strength was distributed over a narrow range of values regardless of the applied hatch distance. Therefore, we present averaged characteristic strength for the front and the back side bending in Table 3. On average, cutting in water improved the front side strength by 7.2% to 109 ± 8 MPa and back side strength by 10.9% to 93.9 ± 7 MPa, compared with cutting in ambient air.

The front side strength of glass strips cut in ambient air decreased with the hatch. The decrease in strength coincided well with increasing average and mean maximum chipping widths and cut sidewall roughness (at *h* = 2 µm). However, the back side strength of glass strips decreased with the hatch, contradicting the improving cut edge quality at smaller hatch values (average and mean maximum chipping widths) and was only supported with the increasing cut sidewall roughness. Furthermore, the vertical oblong concavities visible in Figure 7 were the largest and most dense at *h* = 2 µm at the front surface, which should cause more drastic losses of front side strength. However, relative strength losses were very similar on both sides of the glass strip. Moreover, the relative strength losses were similar to those cut in water, even though oblong vertical concavities did not form during cutting in water.

In the case of cutting in water, the cut sidewall surface roughness remained constant regardless of the hatch and should not affect the strength of glass strips differently in the investigated *h* range. The mean maximum chipping width decreased with the hatch; therefore, the change in the characteristic strength at the front side of the strips could only be supported by the increase in average chipping width. In the case of back side strength, the maximum value was obtained at the smallest hatch value of 2 µm and was supported with the decrease in average and mean maximum chipping widths.

As a result, we did not observe a relationship between the sidewall roughness measurements or the average and mean maximum chipping widths with the characteristic strength of glass strips at different hatch values.

However, the formation of vertical cracks on the glass strip sidewalls was correlated with characteristic strength measurements. According to Figure 7, the cracks were short, formed between ridges and were mostly discontinuous in both cutting environments at the optimal hatch value (*h* = 10 µm), resulting in the highest characteristic strength. However, the length and density of cracks increased with the decreasing hatch, indicating the loss of strength in both environments. Thus, we believe that the evolution of the crack network at the cut sidewall is mostly responsible for the characteristic strength degradation of strips cut at smaller hatch distances than the optimal.

Furthermore, we speculate that the characteristic strength of glass strips cut in water was higher due to more efficient cooling. Laser cutting generates high temperature gradients, causing significant stress fields in the ablation zone [58]. Therefore, better cooling lessens the heat diffusion into the material, as well as the generated stresses [8], consequently decreasing the crack depth and increasing the mechanical strength of samples [52].

### 3.2. High-Laser-Power Cutting Regime

Scaling up the manufacturing throughput is one of the major objectives for successfully implementing direct laser ablation technology for glass cutting applications. Considering that the direct ablation is much more energy-demanding than other laser-based glass cutting techniques (such as internal scribing and rear side ablation), significantly higher average laser power should be used to keep the cutting speed and material removal rates competitive. Unfortunately, glass is a brittle material with low heat conductivity, limiting the practical use of the full potential of high-power lasers. As mentioned in Section 3.1.1, glass plates used in the experiment could not withstand incident laser power above 4 W and shattered during the cutting. Fortunately, water ensured sufficient cooling, enabling glass to be cut into smaller strips at higher laser power.

For this reason, additional high-power glass cutting experiments were performed in water at an incident laser power of 15.5 W. The laser beam spot size was increased to 30 µm by removing the beam expander from the laser beam optical path. This was performed to maintain optimal laser fluence at maximum laser power while staying in the lasers’ operating pulse repetition rate range. Cutting width was not changed (300 µm) to keep experimental conditions similar to that in the low-power cutting regime. Other laser processing parameters, such as pulse repetition rate, hatch and laser beam scanning speed, were re-optimised for larger beam width and higher average laser power.

The optimised processing parameters for cutting glass in a high-power regime are presented in Table 4. At 15.5 W, the borosilicate glass ablation efficiency was 8.7 µm^3^/µJ, giving an effective cutting speed of 1.8 mm/s, whereas the effective cutting speed per incident laser power was 0.116 mm/s/W. Compared with the low-power cutting regime in water, ablation efficiency and effective cutting speed per incident laser power increased by 16% and 29%, respectively. The increase in both parameters was associated with the more pronounced glass cracking and disintegration at higher laser power. However, the optimal laser fluence remained unchanged (6.1 J/cm^2^).

For the sake of brevity, in further text, only the cuts produced at the optimal hatch will be discussed.

**Figure 17:**
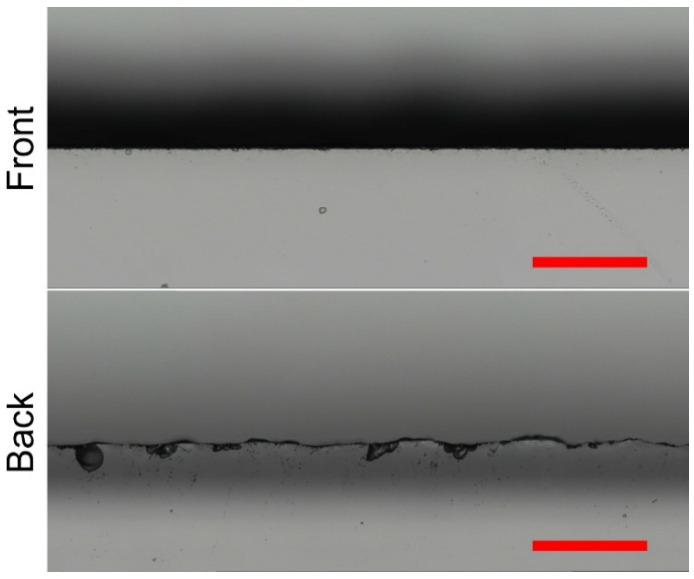
Optical micrographs showing front and back surface cut edges. Cutting was performed in water at 15.5 W. The scale bars represent 200 μm and apply to all panels in the figure.

The micrographs of the front and back surface cut edges produced in water at 15.5 W are presented in Figure 17. The average chipping and mean maximum chipping widths at the front surface were 1.5 ± 0.7 µm and 20 ± 19 µm, respectively. Both parameters doubled in the high-laser-power cutting regime, compared with cutting in water at 2.75 W. Despite this, the average chipping width remained almost three times smaller compared with cutting in ambient air at 2.1 W.

At the bottom surface, higher incident laser power had almost no effect on the average chipping width, which increased only by 3% to 7.1 ± 4 µm, compared with glass cutting in water at 2.75 W. The mean maximum chipping width in the high-power regime was 30 ± 10 µm. Compared with cutting at low laser power in water and ambient air, the mean maximum chipping widths decreased by 32% and 26%, respectively.

**Figure 18:**
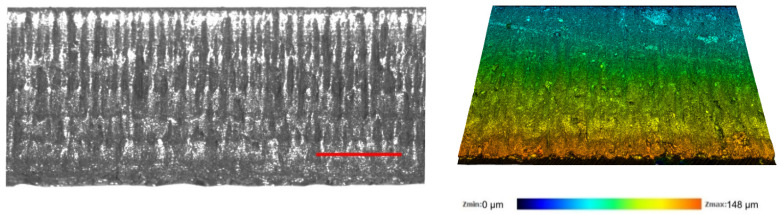
Optical micrograph (**left**) and the topography (**right**) of the cut sidewall produced in water at 15.5 W. The scale bar in the left panel represents 200 µm.

The 3D topology and the optical micrograph of the cut wall produced at 15.5 W are shown in Figure 18. The micrographs revealed that the sidewall was covered with vertical notches. The direction of notches was normal to the glass surface, the same as the direction of cracks formed in glass strips in Figure 7. According to the micrographs, some notches were long and could span between several ridges, whereas the length of others was limited to the period of ridges. The variation in length was similar to vertical cracks formed in glass strips cut in ambient air at low average laser power (see Figure 7). Thus, we believe that the notches were formed during the coalescence of adjacent cracks during the laser ablation at higher incident power.

The sidewall remained covered with periodically recurring ridges, as in the strips cut under the low-power cutting regime (Figure 18). The period of ridges corresponded well with the relationship between the cut sidewall taper angle and the hatch distance given in Section 3.1.2. The ridges could be easily identified in the optical micrograph. However, they were difficult to distinguish in topographical images, indicating that the ridge height was similar to the surface roughness of the cut wall, which was 0.82 µm. Compared with the low-power cutting regime in water, the *R_a_* increased by 20%. The formation of notches at the cut sidewall seen in Figure 18 was primarily responsible for the increase in surface roughness.

Finally, bending tests were applied on laser-cut glass strips, and the tension was applied only on the stronger (front surface) side of the glass strips. The obtained average characteristic strength was 107.5 ± 6.6 MPa. According to the results, the strength of glass strips cut at 15.5 W decreased by 8.5% compared with strips cut at 2.75 W in water, and almost equally with the strength of glasses cut in ambient air at 2.1 W. However, the glass strips were broken more predictably in the high-power cutting regime than at 2.75 W: the shape parameter of strips cut at 15.5 W was 9.2 ± 0.9, whereas at 2.75 W, the shape parameter was 4.8 ± 0.6.

## 4. Conclusions

In this study, we investigated borosilicate glass cutting in ambient air and water with 355 nm wavelength picosecond laser pulses. Laser pulse repetition rate, fluence, beam scanning speed and hatch parameters were optimised to cut 400 µm thick glass plates into 26 × 6 mm^2^ strips under efficient ablation conditions in both environments. Cutting under optimised laser parameters in a low-laser-power regime (2.1 W in ambient air and 2.75 W in water) was more efficient in water (7.5 µm^3^/µJ) than in ambient air (6.6 µm^3^/µJ), by 14%. Nevertheless, the effective glass cutting speed per incident laser power remained similar in both cutting environments (0.095 mm/s/W in water and 0.09 mm/s/W in ambient air). The reason for the discrepancy between ablation efficiency and effective cutting speed per incident power was mainly caused by the laser cut taper angle, which was shallower in water. As a result, steeper cut walls in water contributed to a higher ablation efficiency, but had little effect on the thickness of the removed material layer per single laser scan.

Additionally, more efficient cooling in water allowed us to cut glass at the maximum average laser power of 15.5 W, whereas glass in ambient air fractured when the incident laser power exceeded 4 W. As a result, effective glass cutting speed increased from 0.26 mm/s (at 2.75 W) to 1.8 mm/s (at 15.5 W) in water. Additionally, at higher incident laser power, the ablation efficiency increased by 16% to 8.7 µm^3^/µJ and effective glass cutting speed per incident laser power increased to 0.116 mm/s/W. Improved ablation efficiency and effective cutting speed were associated with the increased cracking and disintegration of brittle glass material at higher power levels.

Under the optimal cutting parameters (in terms of ablation efficiency), glass strips cut in the water had the highest characteristic strength: 117.6 MPa when bending was applied from the front side and 107.3 MPa from the back side of laser cut glass strips. In comparison, glass strips cut in ambient air had a lower characteristic strength on both sides (107.3 MPa and 88.9 MPa at the front and back surface sides, respectively). The average front side surface characteristic strength of samples cut at 15.5 W in water was 107.5 MPa. The strength of strips in the high-power cutting regime degraded but remained slightly higher than strips cut in ambient air at 2.1 W.

The variation in the hatch had the largest impact on the visual quality of the cut sidewall due to the formation of ridges, ablation efficiency and the characteristic strength of glass strips. The formation of ridges at the cut sidewall was prominent at the optimal hatch value (10 µm). The height and the period of ridges decreased with the hatch until ridges could not be distinguished any more (*h* = 2 µm in ambient air and *h* = 4 µm in water), improving the sidewall quality. However, this negatively affected the ablation efficiency and overall strength of glass strips. Ablation efficiency and strength losses were associated with overheating the glass plate, because the heat accumulation increased with decreasing spacing between the neighbouring cut lines (hatch). The decrease in characteristic strength was associated with the formation of vertical cracks on the cut sidewalls. Micrographs revealed that at the optimal hatch distance, the cracks were short and discontinuous. However, as the hatch decreased, the length of cracks notably increased, spanning between multiple ridges.

The experimental results show that the borosilicate cutting in water was superior to the ablation in ambient air, and represents a promising candidate for wider applications in the industrial cutting of high-quality glass parts.

## Figures and Tables

**Table 1:** Optimised glass cutting parameters (low-laser-power cutting regime).

Cutting Environment	Average Laser Power, W	Ablation Efficiency, µm^3^/µJ	Fluence, J/cm^2^	Scan Speed, mm/s	Pulse Energy, μJ	Pulse Repetition Rate, kHz	Hatch, µm	Cut Width, µm
Air	2.1	6.6	3.5	800	3.2	653	10	300
Water (low power)	2.75	7.5	6.1	600	5.2	529	10	300

**Table 2:** Average chipping widths.

	Cutting in Ambient Air			Cutting in Water		
Hatch, μm	2 μm	6 μm	10 μm	4 μm	7 μm	10 μm
Front surface, μm	6.4 ± 3.7	6.2 ± 2.9	4.3 ± 1.8	0.85 ± 0.4	0.8 ± 0.4	0.75 ± 0.35
Back surface, μm	3.9 ± 2.9	5.4 ± 3.2	6.7 ± 3.3	4.4 ± 2.2	6.7 ± 2.6	6.9 ± 4.3

**Table 3:** Average characteristic strength of glass strips cut in ambient air and water for the front and back side bending cases. Strength was averaged in terms of hatch distance.

Cutting Environment	Front Side	Back Side
Ambient air	101.7 ± 6 MPa	84.7 ± 7 MPa
Water	109 ± 8 MPa	93.9 ± 7 MPa

**Table 4:** Optimised glass cutting parameters. High-laser-power cutting in water.

Cutting Environment	Average Laser Power, W	Ablation Efficiency, µm^3^/µJ	Fluence, J/cm^2^	Scan Speed, mm/s	Pulse Energy, μJ	Pulse Repetition Rate, kHz	Hatch, µm	Cut Width, µm
Water (high power)	15.5	8.7	6.1	1250	21.4	725	15	300
